# Supplementary material for: War-related vicarious trauma among healthcare providers in the war-torn tigray, Northern Ethiopia
Source: PLoS One. 2026 Mar 4;21(3):e0340978. doi: 10.1371/journal.pone.0340978 (PMC12959660; doi:10.1371/journal.pone.0340978)
Supplement: Attachment — Submitted filename: Revision of PLOS One manuscript-Vicarious trauma.docx [file pone.0340978.s002.docx]

**Date: November 12, 2020**

Dear Editor,

I hereby submit a review report of the manuscript, entitled ‘War related -Vicarious Trauma among Healthcare providers in the War-Torn Tigray, Northern Ethiopia.”. I acknowledge the efforts of the authors for performing a good job and writing evidence which is highly needed. Since, it needs further amendment, I suggested to be accepted with Major revision. I believe, this manuscript will be very helpful in passing better evidence for decision making in rebuilding the human resource development.

For the sake of simplicity, it would be good to create line numbers of the manuscript which would be easier for the authors to trace the comments and revised changes.

Abstract:

On the method section of the abstract a statement is described as “VT was assessed using a 7-item standard tool with likert scale…”, **a phrase in a new line statement shall be written with full words of the phrase, otherwise, authors are advised to write Vicarious Trauma instead of VT.**

On the second line of the result section of the abstract, authors need to rephrase the “was found to be”.

The conclusion of the abstract seems overlooked.

**Introduction**

**On the first and second paragraphs:** Authors have verified about the war in Tigray and its effects on the vulnerability of the health workers referring three peer reviewed articles. **Ample literature written by humanitarian organizations have proven the health workforce traumatic experiences, so authors are requested to justify their statements from the perspective of humanitarian agencies.**

**Third and fourth paragraph,** the piece of evidences from Gaza, Poland, Eastern Maynemar, Kenya, USA, Japan and findings from University of Ottawa needs to be consolidated, refined and synthesized. At this level, it would be good if the information could convey precise and concise message, otherwise it would be redundant. Authors are requested to revise the paragraph in a suitable manner for readers.

Though large number of health workforce population and civilians suffer in SSA countries due to war and conflict, **the experiences and prevalence of vicarious trauma in sub Saharan African countries seem to be overlooked. So, authors are requested to provide SSA countries experiences.**

On top of these comments, authors are advised to revise the flow and coherence of the paragraphs in the introduction section. It would be advisable to follow the writing style from global to local and general to specific.

Last paragraph of the introduction section: Authors noted “Despite our personal observations and experiences …….. **the scope of the problem is not investigated**”. The highlighted phrase seems overstated. Because, significant number of report reviews have been documented by MSF,

So, authors are advised to revise the phrase; instead of “not investigated” “the scope of the problem is not adequately investigated” might be an option. It would also be good to rephrase the valid evidence stated as “Except for the presence of vicarious trauma among the medical staff in the hemodialysis unit of the Ayder referral hospital published recently”.

Methods and materials

Study area

It is good that authors noted the number of health facilities and health workforce before the war. However, it seems inadequate information for this manuscript. On top of the information given the categories of the health workers, their speciality and the development of the health workforce and its contribution on the health outcome need to be highlighted.

Study design

The design is not only for the purpose of recruiting healthcare providers. It works for conducting the overall study. **Authors are advised to omit the phrase “recruiting healthcare providers”.**

**Sample Size and Sampling Procedure**

What was the reason for authors to choose simple random sampling technique after using multistage stratified sampling technique, while systematic random sampling technique is the easiest technique? It is from curiosity.

Authors noted “Twelve health professionals fluent in the local language (Tigrigna) were recruited.

Results

**Socio-demographic characteristics of study participants**

The first line statement reads “From the total of 2374 sampled healthcare providers, 2366 participants were included in the study”, I suggest to be corrected as **“of 2374 participants, 2366 participated in the study” Authors are advised to revise their reports to be concise.**

The first paragraph of the result section is with redundant phrases “two third is stated four times in a single paragraph”. **Authors are advised to avoid redundant phrases and rephrase their result statements with eye-catching ideas to readers.**

**Besides authors interpreted “**Nearly two-thirds of the study participants were from the Mekelle and Central zones” that mismatches with the figure on table 1 which is calculated as 26.7% for Mekelle and 15% for Central in total 41.7%. Authors are advised to make correction of the aforementioned result statement.

What standard do you for perceived economic classification?

**In your result section table 2, the result of the odds ratio 0.7 [0.5, 0.9] by zone showed the protective effect of vicarious trauma among health workers in south East zone, do you have any clarification? Can you see back your data again for possible correction of the analysis?**

**Discussion**

**In the first paragraph of the discussion the word those is repeatedly stated, so authors are requested to avoid redundant words and revise the wording.**

The comparison of similarities and variations in the discussion section is more skewed to the Western countries (USA, Europe and Canada). **In order to make careful comparisons with sub-Saharan African countries, Authors need to revisit the discussion section.**

**It would be good if authors made an attempt to align some of their justifications with peer literature review.**

**Authors stated “**Consequently, medical personnel were forced to treat a high volume of patients with the potentially dangerous physical and psychological effects of the conflict on a regular basis”. This statement is not clear. **Is it part of the finding of this study, if not, Authors are requested to put a reference.**

Authors stated “….. many medical experts and **dangerous medical supplies** needed for treatment and therapy have been forced to leave underfunded”, “What does **dangerous medical supplies mean**?” Authors are advised to provide clear statement on the highlighted phrase.

**Many of the justifications stated on the discussion section lack references. Similar justifications are documented in various reports by humanitarian organizations including *MSF, OCHA, ICRC and World Peace foundation led by Professor Alex Dwall, so authors are advised to use the references for their justifications***

**Authors are duly requested to revisit the discussion section for further elaboration and amendment.**

**For the betterment of the manuscript, language revision is highly needed by native speakers.**

**Decision – Major revision**
