# Supplementary material for: War-related vicarious trauma among healthcare providers in the war-torn tigray, Northern Ethiopia
Source: PLoS One. 2026 Mar 4;21(3):e0340978. doi: 10.1371/journal.pone.0340978 (PMC12959660; doi:10.1371/journal.pone.0340978)
Supplement: Attachment — Submitted filename: Plos One review.docx [file pone.0340978.s003.docx]

# War related-Vicarious Trauma among Healthcare providers in the War-Torn Tigray, Northern Ethiopia.

Hagos Degefa Hidru^1*^, Mengistu Hagazi Tequare^2,1^,Abadi Kidanemariam Berhe^3,1^, Gidey Gebremeskel Kidane^4^, Yemane Gebremariam Gebre^5^, Mohamedawel Mohamednigus Ebrahim^4,^, Aregawi Gebreyesus Belay^2^, Gebregziabher Berihu Gebrekidan^2^, Reiye Esayas Mengesha^4^, Gebrekiros Gebremichael Meles^2^, Assefa Ayalew Gebreslassie^2^, Bereket Berhe Abreha^4^, Desalegn Massa Teklemichael^2^, Berihu Gidey Aregawi^3^, Haftom Tesfay Gebremedhin^6^, Gebremedhin Gebreegziabher Gebretsadik^3^, Yemane Berhane Tesfau^3^, Gebreyesus Elfu Mezgebe^7^, Zemichael Weldegebriel Asreshey^8^, Girmay Medhin^9^, Hailay Gebretnsae^1^, Yibrah Alemayehu Haile^10^, Tedros Gobezay Desta^10^, Tsegay Berihu Tesfay^10^, Alem Gebremariam^3,1^

^1^Tigray Health Research Institute, Mekelle, Tigray, Ethiopia.

^2^School of Public Health, College of Medicine and Health Sciences, Mekelle University, Mekelle, Tigray, Ethiopia

^3^Department of Public Health, College of Medicine and Health Sciences, Adigrat University, Adigrat, Tigray, Ethiopia

^4^School of Medicine, College of Health Sciences, Mekelle University, Mekelle, Tigray, Ethiopia

^5^ School of Medicine, College of Medicine and Health Sciences, Adigrat University, Adigrat, Tigray, Ethiopia

^6^Department of psychiatry, College of Medicine and Health Sciences, Adigrat University, Adigrat, Tigray, Ethiopia.

^7^School of Medicine, College of Medicine and Health Sciences, Aksum University, Aksum, Tigray, Ethiopia

^8^School of Public Health, College of Medicine and Health Sciences, Aksum University, Aksum, Tigray, Ethiopia

^9^Aklilu Lemma Institute of Pathobiology, Addis Ababa University, Addis Ababa, Ethiopia

^10^Tigray Health Bureau, Mekelle, Tigray, Ethiopia

^*^ Corresponding author: Email: [hagosdeg@gmail.com](mailto:hagosdeg@gmail.com)

**Abstract**

**Background:** The Tigray War, which started in November 2020, has doubled the burden on healthcare professionals, causing both direct violence and indirect effects as they care for trauma survivors. Due to the lack of evidence regarding the level of vicarious trauma (VT) in this population, we assessed the extent of VT among healthcare providers working in the war-torn Tigray region of Ethiopia.

**Method:** A health facility**-**based cross sectional study design was used to recruit 2,374 healthcare providers from August to September 2023 and the study participants were recruited using stratified random sampling techniques. Data were collected using the Open Data Kit (ODK) and exported to SPSS version 23.0 for analysis. VT was assessed using a 7-item standard tool with likert scale responses ranging from very often (5) to rarely (0). The total score from the responses on these 7 items was categorized as low risk (0–14), moderate risk (15–21), high risk (22-28), and extremely high risk (29–35). Ordinal multivariable logistic regression was used to identify the association of various factors with vicarious trauma. Statistical significance was reported whenever the 95% confidence interval for the odds ratio did not include one and the p-value was less than 0.05.

**Result:** The prevalence of moderate and above VT was found to be 81.6% (95% CI: 80.0, 83.1). Specifically, the burden was found to be 32.4%, 33.3%, and 15.8% for moderate, high levels, , extremely high levels of VT, respectively with significant variability across the administrative zones of the region. The risk of VT increases with increasing age, having a larger family size, among those who live far from their work place, and among those in leadership positions.

**Conclusion:** Healthcare providers in the Tigray region have experienced high level of vicarious trauma, and it has a risk of restricting the overall healthcare service if not properly managed. This calls for a quick collaborative effort to address the condition.

**Key word:** Vicarious trauma, healthcare provider, war-torn, Tigray

# Introduction

Vicarious traumatization (VT) refers to harmful changes that occur in professionals’ views of themselves and others, as a result of exposure to the graphic and/or traumatized psychological needs, beliefs, and memory systems of their clients who treat trauma survivors (1,2). The war that broke out in Tigray, Northern Ethiopia, in November 2020 has resulted in severe human and material damages. It has costed the physical, mental, economic, and psychological health of the people in Tigray in general and the healthcare providers in particular (3, 4).

Healthcare providers were burdened by both the direct effects of the war on them and the indirect effects of the conflict, which forced them to care for those who were hurt by the war and work with trauma survivors (5). The deliberate targeting of the war on the health care system is causing a total disruption of the health care services. The effect of conflicts provided a prominent example of this experience (4). Health care providers are particularly vulnerable to vicarious trauma as a result of repeated exposure to details of clients’ trauma experiences (6).

Research showed that the more time health professionals spend with their traumatized clients and the heavier the workload, the higher the providers’ risk of developing vicarious trauma. For instance, during the battle on Gaza, 45% of the health professionals had vicarious trauma (7). A report from Poland showed that 43.4% of health care providers had developed secondary trauma (8). Similarly, a study conducted by the University of Ottawa reported that 50% of therapists who worked with sexual offenders had vicarious trauma (9). A systematic review revealed that 56% of health care providers had developed secondary trauma (10). A study conducted in eastern Maynemar indicated that healthcare providers in a conflict without a supportive work environment were at greater risk for vicarious trauma (11).

Vicarious trauma (VT) continues to be prevalent among healthcare providers, although the rates vary. A study from Kenya revealed that for 67% of healthcare providers working at hospitals, vicarious trauma is a real threat (12). A study conducted in the USA revealed that 12% of palliative care physicians reported vicarious trauma and depressive symptoms (13). A systematic review study showed that vicarious trauma rates ranged from as low as 26% to as high as 90% across various clinical settings (14). Additionally, a study carried out in the USA among child welfare professionals indicated that about 26%-35% of participants experienced vicarious trauma (15). Furthermore, a study conducted among nurses in Japanese hospitals found a secondary trauma prevalence of 90.3% (16).

Despite our personal observations and experiences with such trauma among healthcare providers, in the war-torn Tigray region, the scope of the problem is not investigated. Except for the presence of vicarious trauma among the medical staff in the hemodialysis unit of the Ayder referral hospital published recently (5). Thus, there are many other consequences of war and conflict that need thoughtful consideration. Among these, the burden on health care providers and the extremely serious lack of access to medical supplies to treat survivors that often result from the war cannot be over-emphasized. Therefore, this facility-based cross-sectional study was conducted to estimate the extent of vicarious trauma among healthcare professionals in the Tigray region of Ethiopia.

# Methods and Materials

## **Study Area and settings**

The study was conducted in a war-torn Tigray region of Ethiopia. Before the breakout of the war, the region had a well-structured three-tiered health system; a Primary Healthcare Unit that includes health posts, health centres and primary hospitals; secondary care provided by general hospitals; and tertiary care provided by specialized referral hospitals (17). As of November 2020, the region had 40 hospitals (2 specialized referral, 14 general, 24 primary), 226 health centers, and 741 health posts, which staffed over 25,000 health care providers who provided a variety of health care services (18, 19) (Figure 1).

Figure 1: Geo-map of the selected health facilities in Tigray, 2023.

## **Study Design and Period population?**

A facility-based cross-sectional study design was used to recruit health care providers working in the war-torn Tigray region of Ethiopia from August to September 2023.

## **Sample Size and Sampling Procedure**

The sample size was estimated considering a confidence level of 95% (Z= 1.96), a margin of error of 0.03, a 50% prevalence of VT among healthcare workers, a 10% non-response rate and a design effect of 2. This resulted in a final sample size of 2374. A stratified multistage sampling technique was used to reach each health facility. A total of 2374 study participants were proportionally allocated to each of the selected health facilities and then a simple random sampling technique was used to recruit individual potential respondents within the study health facilities.

## **Data Collection Tool and Method**

## A total of seven items from the Crisis and Trauma Resource Institute's (CTRI) tool were used to evaluate vicarious trauma among healthcare providers. The vicarious trauma-related 7-item questionnaire with responses on a Likert scale ranging from very often (5) to rarely (0) was used to measure vicarious trauma. To estimate the degree of vicarious trauma, the scores were added up; larger scores indicate a greater degree of vicarious trauma. The CTRI classifies trauma with a cumulative score as low risk (0–14), moderate risk (15–21), high risk (22-28), and extremely high risk (29–35) (20).

## The data were collected through a face-to-face interviewer-administered questionnaire. After extensive revision of the English questionnaire, the final English version was translated into the local language by language experts. Supervisors and data collectors were trained on the objective of the study, instructions for the method, how to obtain informed consent, how to approach participants, ethical procedures, and general information on vicarious trauma. Twelve health professionals fluent in the local language (Tigrigna) were recruited.

## **Data Quality Control**

## The questionnaire was pre-tested on 5% of the study population in the non-selected health facilities to check the open data kit (ODK) completeness and to ensure clarity, wording, logical sequence, and skip patterns of the questions. Appropriate modifications were made after discussing them with the supervisor before starting the actual data collection process. Data collection location, accuracy, and completeness were monitored by the data manager by looking at the server-centered. In addition, every day the filled-out questionnaire is checked before a respondent leaves the setting by data collectors and supervisors. The reliability of the item was measured by a Cronbach's alpha that ranged from 0.835 to 0.856.

## **Data Processing and Analysis**

# Data were exported from ODK to SPSS version 23.0 for cleaning and statistical analysis. Descriptive statistical methods such as frequencies and percentages were used to summarize categorical data, while the mean and the standard deviation were used for continuous variables. The association of the independent variables with the level of vicarious trauma (low risk, moderate risk, high risk, and extreme high risk) was investigated using ordinal logistic regression. Variables having p ≤ 0.05 on the bivariate ordinal logistic regression analysis were candidates for the ordinal multivariable logistic regression analysis to control confounders. Statistical significance was reported whenever p-value ≤ 0.05, and the strength of the association was quantified using the adjusted odds ratio (OR) along with a 95% confidence interval. A multi-collinearity test was conducted to see any correlation between the independent variables using the co-linearity diagnostic test (VIF and tolerance test). The model assumptions for goodness of fit were checked and fitness was declared at p-value ≥ 0.05. When we report our results of multivariable ordinal logistic regression, we used the term “higher level vicarious trauma,” which means extreme high than combined low, medium, and high; or combined “high and extreme high” than combined low and medium; or combined “medium, high, and extreme high’’ than low.

# Result

## **Socio-demographic characteristics of study participants**

From the total of 2374 sampled healthcare providers, 2366 participants were included in the study, resulting in a response rate of 99.7%, and 60.7% of these respondents were female. The respondents' mean age was 34 years (SD=±8) and more than 80% were within the age range of 20 to 39 years. Two-third of the study participants (65.0%) were married, and about two-thirds had more than three family members, lived with their spouses, and had no own house. The majority (96.4%) of the study participants were followers of the orthodox religion. Nearly two-thirds of the study participants were from the Mekelle and Central zones, and about three-fourths worked in hospital settings. The participants had a median (IQR) work experience of 8.0 (5.0, 12.0) years and a median annual income (IQR) of 8017 Ethiopian Birr (range: 7071.0–9056.0). Two-thirds of the respondents, 66.1%, were living with their parents. Almost half (48.0%) of the respondents were nurses, and about 1452 (61.4%) of the respondents had a BSc degree. The majority, 94.3% of the study participants, had no alternative source of income, and more than half perceived themselves as economically poor. More than three-fourths (79.0%,),reported working more than or equal to 40 hours per week, and more than half, 54.1%, occasionally worked on weekends. About half (50.4%), of the healthcare providers took less than 25 minutes to reach their working health facilities (Table 1).

*Table 1: Socio-demographic and work-related characteristics of the study participants in Tigray, Ethiopia, from August to September 2023.*

| **Variables (N=2366)** | **Categories** | **Frequency** | **Percentage (%)** |
| --- | --- | --- | --- |
| **Zone address** | Mekelle | 631 | 26.7 |
|  | Central | 356 | 15.0 |
|  | Eastern | 701 | 29.6 |
|  | North-west | 254 | 10.7 |
|  | South-east | 265 | 11.2 |
|  | South | 159 | 6.7 |
| **Age (in year)** | 20-29 | 864 | 36.5 |
|  | 30-39 | 1052 | 44.5 |
|  | 40-49 | 211 | 8.9 |
|  | 50+ | 239 | 10.1 |
| **Sex of the respondents** | Male | 930 | 39.3 |
|  | Female | 1436 | 60.7 |
| **Marital status** | Never married | 666 | 28.1 |
|  | Currently married | 1538 | 65.0 |
|  | Divorced/Widowed/Separated | 162 | 6.8 |
| **Family size** | 1-2 | 694 | 29.3 |
|  | 3-4 | 999 | 42.2 |
|  | ≥5 | 673 | 28.4 |
| **Permanent residence of family members** | Living together | 1565 | 66.1 |
|  | Living separately | 801 | 33.9 |
| **Have your own house** | Yes | 678 | 28.7 |
|  | No | 1688 | 71.3 |
| **Type of living house** | Rent house | 1661 | 70.2 |
|  | Own house | 450 | 19.0 |
|  | Family house | 165 | 7.0 |
|  | Governmental house | 90 | 3.8 |
| **Perceived economic classification** | Rich | 8 | 0.3 |
|  | Medium | 912 | 38.5 |
|  | Poor | 1342 | 56.7 |
|  | Unable to classify | 79 | 3.3 |
|  | I do not want to talk | 25 | 1.1 |
| **Current profession** | Laboratory | 160 | 6.8 |
|  | Pharmacy | 175 | 7.4 |
|  | Medical doctor | 182 | 7.7 |
|  | Nurse | 1135 | 48.0 |
|  | Midwife | 297 | 12.6 |
|  | Public health officer | 120 | 5.1 |
|  | Health Extension worker | 145 | 6.1 |
|  | Others | 152 | 6.4 |
| **Educational level** | Level 3/4 or Diploma | 645 | 27.3 |
|  | BSc Degree | 1452 | 61.4 |
|  | MSc/MPH | 87 | 3.7 |
|  | Medical Doctor | 98 | 4.1 |
|  | Specialist/Sub-specialist | 84 | 3.6 |
| **Managerial**  **position** | Yes | 441 | 18.6 |
|  | No | 1925 | 81.4 |
| **Total work experience** | <5 years | 1036 | 43.8 |
|  | 5 to 9 years | 952 | 40.2 |
|  | 10 years or above | 378 | 16.0 |
| **Alternative source of income** | Yes | 134 | 5.7 |
|  | No | 2232 | 94.3 |
| **Work at weekends** | Always | 652 | 27.6 |
|  | Sometimes | 1279 | 54.1 |
|  | Rarely | 130 | 5.5 |
|  | Not at all | 305 | 12.9 |
| **Work at night shift** | Always | 298 | 12.6 |
|  | Sometimes | 1431 | 60.5 |
|  | Rarely | 115 | 4.9 |
|  | Not at all | 522 | 22.1 |
| **Means of travel to work** | Taxi/Bajaj/Public Transport | 236 | 10.0 |
|  | Facility car | 351 | 14.8 |
|  | On foot | 1737 | 73.4 |
|  | Others | 42 | 1.8 |
| **Distance from home to workplace in minutes** | <=25 | 1192 | 50.4 |
|  | 26-50 | 691 | 29.2 |
|  | 51-75 | 239 | 10.1 |
|  | >=76 | 244 | 10.3 |
| **Monthly gross salary in ETB** | =<7071 | 1036 | 43.8 |
|  | 7072-9056 | 1018 | 43.0 |
|  | >=9057 | 312 | 13.2 |
| **Working hours per week** | <40 hours | 1550 | 65.5 |
|  | >=40 hours | 816 | 34.5 |

## **Prevalence of Vicarious Trauma**

The overall prevalnce of vicarious trauma (moderate and above) was found to be 81.6% [95% CI: 80.0, 83.1], with their specific level indicated as moderate, high, and extremely high accounting for 32.4%, 33.3%, and 15.8%, respectively (Figure 2).

*Figure 2: Level of vicarious trauma among health care professionals in the war-torn Tigray, North Ethiopia, August to September, 2023.*

## **Factors Associated with Vicarious Trauma**

Eight variables (zone, age category, family size, management position, working hours, and walking distance to the health facility) were included in the multivariable ordinal logistic regression model, and all variables, except working hours, were found to be significantly associated with vicarious trauma.

There was a higher level of vicarious trauma among healthcare providers working in the north-west (AOR = 1.5; 95% CI: 1.1, 2.0) and central (AOR = 1.4; 95% CI: 1.1, 1.7) zones compared to the Mekelle zone. Compared to healthcare providers in their age groups of 20–29 years, the odds of being at a higher level of vicarious trauma were higher as age increased from 30-39 years (AOR = 1.3, 95% CI: 1.1, 1.6), 40–49 years (AOR = 1.5, 95% CI: 1.1, 2.0), and 50 years and older (AOR = 2.3, 95% CI: 1.7, 3.0). The odds of being at a higher level of vicarious trauma were 1.5 times higher among those with management positions as compared to healthcare providers who were not in management positions (AOR = 1.5, 95% CI: 1.2, 18). The odds of experiencing a higher level of vicarious trauma were 1.2 times higher among healthcare providers with family sizes of five and above as compared to those with family sizes of less than five (AOR = 1.2, 95% CI: 1.1, 1.5).

The likelihood of experiencing a higher level of vicarious trauma was 1.3 times higher among healthcare providers walking a distance greater than 60 minutes from home to their health facility than healthcare providers whose walking distance is less than 30 minutes (AOR = 1.3, 95% CI: 1.1, 1.6) (Table 2).

*Table 2: Factors associated with vicarious trauma among healthcare providers in Tigray, Ethiopia, August to September, 2023.*

| **Independent Variables (N=2366)** | | **Vicarious trauma, n (%)** | | | | **COR [95% CI]** | **AOR [95% CI]** |
| --- | --- | --- | --- | --- | --- | --- | --- |
|  |  | **Low** | **Medium** | **High** | **Extreme high** |  |  |
| **Zone address** | Mekelle | 129 (18.4) | 251 (35.8) | 225 (32.1) | 96 (13.7) | Ref. | Ref. |
|  | Central | 99 (15.7) | 179 (28.4) | 215 (34.1) | 138 (21.9) | 1.5 [1.2, 1.8] | **1.4 [1.1, 1.7] ^b^** |
|  | Eastern | 73 (20.5) | 104 (29.2) | 120 (33.7) | 59 (16.6) | 1.1 [0.9, 1.4] | 1.0 [0.8, 1.3] |
|  | North west | 25 (9.8) | 65 (25.6) | 105 (41.3) | 59 (23.2) | 2.0 [1.6, 2.6] | **1.5 [1.1, 2.0] ^a^** |
|  | South east | 40 (25.2) | 63 (39.6) | 49 (30.8) | 7 (4.4) | 0.6 [0.5, 0.9] | **0.7 [0.5, 0.9]** **^a^** |
|  | South | 70 (26.4) | 105 (39.6) | 74 (27.9) | 16 (6) | 0.6 [0.5, 0.8] | 0.8 [0.6, 1.0] |
| **Age in years** | 20-29 | 206 (23.8) | 299 (34.6) | 265 (30.7) | 94 (10.9) | Ref. | Ref. |
|  | 30-39 | 174 (16.5) | 347 (33.0) | 355 (33.7) | 176 (16.7) | 1.5 [1.28, 1.77] | **1.3 [1.1, 1.6] ^b^** |
|  | 40-49 | 37 (17.5) | 57 (27) | 77 (36.5) | 40 (19.0) | 1.7 [1.30, 2.27] | **1.5 [1.1, 2.0] ^b^** |
|  | 50+ | 19 (7.9) | 64 (26.8) | 91 (38.1) | 65 (27.2) | 2.8 [2.19, 3.70] | **2.3 [1.7, 3.0]^c^** |
| **Family size** | 1-4 | 342 (202) | 561 (33.1) | 559 (33.0) | 231 (13.6) | Ref. | Ref. |
|  | ≥ 5 | 94 (14.0) | 206 (30.6) | 229 (34.0) | 144 (21.4) | 1.5 [1.29, 1.79] | **1.2 [ 1.1, 1.5]^a^** |
| **Management position** | Yes | 75 (17.0) | 109 (24.7) | 162 (36.7) | 95 (21.5) | 1.5 [1.22, 1.79] | **1.5 [1.2, 1.8]^c^** |
|  | No | 361 (18.8) | 658 (34.2) | 626 (32.5) | 280 (14.5) | Ref. | Ref. |
| **Working hour** | <40 | 273 (17.6) | 482 (31.1) | 528 (34.1) | 267 (17.2) | Ref. | Ref. |
|  | ≥ 40 | 163 (20.0) | 285 (34.9) | 260 (31.9) | 108 (13.2) | 0.8 [0.68, 0.92] | 1.0 [0.8, 1.1] |
| **Walking distance** | <30 | 252 (21.1) | 387 (32.5) | 387 (32.5) | 166 (13.9) | Ref. | Ref. |
|  | 30-59 Minute | 111 (15.9) | 229 (32.9) | 250 (35.9) | 107 (15.4) | 1.2 [1.05, 1.46] | 1.1 [0.9, 1.3] |
|  | 60+minutes | 73 (15.3) | 151 (31.7) | 151 (31.7) | 102 (21.4) | 1.4 [1.19, 1.75 | **1.3 [1.1, 1.6] ^a^** |
| AOR = Adjusted Odds Ratio, COR = Crude Odds Ratio CI = Confidence Interval  a=p <0.05, b=p <0.01, c=p<0.001 | | | | | | | |

# Discussion

This study assessed the prevalence of vicarious trauma among healthcare providers in the war-torn Tigray region using the Crisis and Trauma Resource Institute's (CTRI) classification with a cumulative score of low risk (0–14), moderate risk (15–21), high risk (22-28), and extremely high risk (29–35). More than eighty percent of the healthcare providers had VT, and it was significantly higher among those of older age, those with a larger family size, those walking sixty minutes and above to their work place, those with managerial positions, and those with variation across working zones.

The prevalence of VT (81.6%) in our study revealed a high number of healthcare providers suffering from moderate and severe vicarious trauma. This finding was higher than studies done in Kenya (67%) (15), Arizona (61.3%) (22), the southern United States (70%) (23), Gaza (45%) (7), Poland (43.4%) (8), the United States of America (12%) (14), (35%) (24), and Canada (50%) (9). This disparity may have resulted from the severity of the war in Tigray, which raised the risk of violence and was characterized by great cruelty, reports of killings, heinous sexual violence, and immense health needs created by the conflict. Consequently, medical personnels were forced to treat a high volume of patients in a regular basis. Another explanation could be that many medical experts and dangerous medical supplies needed for treatment and therapy have been forced to leave underfunded, damaged, and robbed healthcare institutions. This places a great deal of strain on the remaining employees, who are already probably overburdened and worn out, making them even more susceptible to vicarious stress. The Tigray war, which was characterized by ethnic violence and the targeting of civilians (ref), which medical professionals may see personally while tending to patients, this could be because the victims have experienced personal loss, have lost loved ones or families, or have been uprooted themselves. This may exacerbate their inability to detach themselves from their traumatized patients and heighten their sense of helplessness and hopelessnes (ref).

The odds of experiencing a higher level of vicarious trauma among healthcare providers were higher as they went from Mekelle to the Central and Northwest zones. This might mean healthcare providers working in these regions were exposed to more **violence and trauma,** heavy fighting during the war, shortages of medical supplies as a result of the war, may have lost colleagues, friends, or family members during the war, **and may** have met and provided care for people who had been injured by the crude troops of Eritrean and Amhara Milasha, like the of mass massacre in Tsion on November 29, 2020. As a result, the looted health system and more traumatized people demanding healthcare providers were high, making them worried.

Similarly, the odds of experiencing a higher level of vicarious trauma were high as the age group increased from 20-29 to 30-39 years, 40-49 years, and 50+ years, which is in line with the study done in Kenya (15). This implication might indicate the life stage and responsibilities; this means care providers over 30 may have additional personal stressors like family obligations, more connectedness and responsibilities to nearby communities, financial concerns, or aging parents, making them less resilient to additional emotional burdens from work in addition to their traumatized clients or patients they met. It might also be because of their **changes in coping mechanisms**; this means that over time, coping strategies used earlier in a career may become less effective (ref), and healthier coping mechanisms may not yet be developed, making individuals more vulnerable to the effects of vicarious trauma (ref).

In addition, the odds of being at a higher level of vicarious trauma were high among healthcare providers with a family size of five or above as compared to those with a family size less than five. The reason might be due to **increased emotional demands (ref). Having** larger families might require juggling work and family responsibilities, potentially leading to stress and fatigue, making it harder to process work-related trauma ref**.** L**imited personal time and** reduced opportunities for self-care and emotional processing due to family commitments can exacerbate the impact of vicarious trauma ref. The odds of having a higher level of vicarious trauma were high among healthcare providers in management positions as compared to those without postion. Managers are likely responsible for the care of their staff as well as patients during the crisis, but being hampered by the limited resources and seriousness of the war might lead to moral distress and feelings of helplessness, which can contribute to vicarious trauma. Additionally, it might be difficult to make decisions about resource allocation, staffing, and patient care under perilous circumstances ref. It might be exposed to a secret broader information about the healthcare system’s such as displaced staff, perilous medical supply, traumatized patients, and witnessing the overall impact of the war on healthcare delivery.

Furthermore, a higher level of vicarious trauma was experienced among healthcare providers who had to walking a distance of sixty minutes or more to their health facility. This might be because of the nature of the war, which was totally besiege and siege ref . As a result, the absence of bank services, the absence of monthly salaries, the ban on any communication, and the lack of fuel due to the war might have forced healthcare providers to travel long distances on foot to reach their facilities ref. This would mean healthcare profesionals see more critically ill patients and those whose conditions worsen due to a lack of timely treatment, effectively adding to their sense of helplessness and frustration and further increasing their emotional burden of vicarious trauma ref. Additionally, walking through war-torn areas could expose healthcare workers to more sights and sounds of the conflict, and witnessing the physical and emotional suffering of the people firsthand could contribute to vicarious trauma.

# Strength and limitation

This study covered public health facilities in all zones of the war-torn Tigray region except the western and part of the southern zones because of security issues and had a large sample size. As limitation in this study, private facilities were not included, and recall bias might be introduced, especially for those affected by an extremely high risk of vicarious trauma.

# Conclusion and recomendation

Eight out of ten healthcare workers in Tigray were found to have experienced vicarious trauma. This rate was found to be significantly higher in those who were older, had larger families, lived farther away from their place of employment, and traveled on foot, and worked in the areas that saw frequent and intense fighting in the central and northwest of the region, which is far from the capital city than the Mekelle Zone. The health workforce's high rate of vicarious trauma may be detrimental to the standard of care provided to the population. Thus, the government ought to act right away to address the underlying causes of the suffering that medical professionals witness, working in tandem with other stakeholders.

# Ethical Consideration

# Ethical approval and clearance were obtained from the Mekelle University Institutional Review Board (IRB) with a reference number (MU-IRB 2049/2023). Then the Tigray Regional Health Bureau (TRHB) secured a letter of cooperation from each of the zonal and district administrations of the selected health facilities. After being informed of the aim of the study by data collectors and supervisors, written consent was obtained from each study participant. Confidentiality of the information is assured, and privacy is respected and kept as well.

# Conflict of interest

The authors declare that they have no conflicts of interest

# Authors Contribution

HDH and AG had involved in the whole process, starting with title selection, design, statistical analysis, and developing the manuscript. All authors were involved in proposal development, data collection, and read and approving the final draft of the manuscript.

# References

1.	Baird K, Kracen AC. Vicarious traumatization and secondary traumatic stress : A research synthesis stress : A research synthesis . 2016;5070(October).

2.	Canfield J. Secondary Traumatization, Burnout , and Vicarious Traumatization : A Review of the Literature as It Relates to Therapists Who Treat Trauma. 75(2):81–101.

3.	Gesesew H, Berhane K, Siraj ES, Siraj D. The impact of war on the health system of the Tigray region in Ethiopia : a response to complaints. 2022;10–2.

4.	Gebrehiwet TG, Abebe HT, Woldemichael A, Gebresilassie K, Tsadik M, Asgedom AA, et al. War and Health Care Services Utilization for Chronic Diseases in Rural and Semiurban Areas of Tigray, Ethiopia. JAMA Netw Open. 2023;6(8):E2331745.

5.	Berhe E, Tesfay B TH. Vicarious trauma on the hemodialysis healthcare workers in the besieged Ethiopia’s Tigray region: a call to action. BMC Med. 2022;Nov 8;20(1.

6.	Sui X PA. Vicarious trauma: The psychological impact of working with survivors of trauma for South African psychologists. J Psychol Afr.

7.	Finklestein M, Stein E, Greene T, Bronstein I, Solomon Z. Posttraumatic stress disorder and vicarious trauma in mental health professionals. Heal Soc Work. 2015;40(2):e25–31.

8.	Gurowiec PJ, Nina Oginska-Bulik ´, Michalska P, And EK dra, Skarbaliene˙ A. The Role of Satisfaction With Job and Cognitive Trauma Processing in the Occurrence of Secondary Traumatic Stress Symptoms in Medical Providers Working With Trauma Victims. Front Psychol | www.frontiersin.org. Volume 12(Article 753173).

9.	Firestone P. The Impact on Therapists Who Work With Sexual Offenders. 2007;8(1):67–83.

10.	Huggard P, Law J, Newcombe D. A systematic review exploring the presence of vicarious trauma, compassion fatigue, and secondary traumatic stress in alcohol and other drug clinicians. Australas J Disaster Trauma Stud. 2017;21(2):65–72.

11.	Lim AG, Stock L, Shwe Oo EK, Jutte DP. Trauma and mental health of medics in eastern Myanmar’s conflict zones: A cross-sectional and mixed methods investigation. Confl Health. 2013;7(1):1–13.

12.	Roberts C, Darroch F, Giles A, van Bruggen R. You’re carrying so many people’s stories: vicarious trauma among fly-in fly-out mental health service providers in Canada. Int J Qual Stud Health Well-being [Internet]. 2022;17(1):1–11. Available from: https://doi.org/10.1080/17482631.2022.2040089

13.	Vachon M.L.S. & Mueller M. Burnout and symptoms of stress. In: Breitbart W, Chochinov HM, eds. . Handb Psychiatry Palliat Med New York, NY Oxford Univ Press.

14.	Musili PM, Sirera AM, Kathungu BM. Vicarious Traumatization among Professional Caregivers and Support Staff in Selected Hospices in Kenya. Int J Soc Sci Econ Invent. 2018;2(07):1–11.

15.	Middleton J. The Relationship Between Vicarious Traumatization and Job Retention among Child Welfare Professionals.

16.	Komachi, M.H, Kamibeppu, K., Nishi, D., Matsuoka Y. Secondary Traumatic Stress and Associated Factors among Japanese Nurses Working in Hospitals. Int J Nurs Pract. (18: 155–163).

17.	TRHB. Tigray regional health bureau II ten years health Bulletin (EFY 1998-2007 or 2006/7-2014/5. Mekele, Tigray, Ethiopia: Tigray Regional Health Bureau (TRHB),. 2016;

18.	Damage Assessment Task Force (Mekelle University and Tigray Regional Health Bureau), Health and Economic Impact of the War on Tigray on IDPs Hosted in Individual Households and Communities. 2022;

19.	Gebregziabher M, Amdeselassie F, Esayas R, Abebe Z, Silvia H, Teklehaimanot AA, et al. Geographical distribution of the health crisis of war in the Tigray region of Ethiopia. BMJ Glob Heal. 2022;7(4):1–11.

20.	Burn out , Compassion fatgue and vicarious trauma assessment. Cris Trauma Resour Inst Inc www.ctrinstitute.com · 1-877-353-3205. :2–3.

21.	Branson. The Relationship between Vicarious Trauma and Sexual Desire in Behavioral Health Clinicians. Arizona ProQuest LLC.

22.	Bride BE. Prevalence of secondary traumatic stress among social workers. Soc Work 52, 63-70 doi 101093/sw/52163. 2007;

23.	Middleton J. The Relationship Between Vicarious Traumatization and Job Retention among Child Welfare Professionals. 2011;
