# Supplementary material for: War-related vicarious trauma among healthcare providers in the war-torn tigray, Northern Ethiopia
Source: PLoS One. 2026 Mar 4;21(3):e0340978. doi: 10.1371/journal.pone.0340978 (PMC12959660; doi:10.1371/journal.pone.0340978)
Supplement: Attachment — Submitted filename: Response to reviewers.docx [file pone.0340978.s006.docx]

**Responses to editor and reviewers’ comments**

Dear Academic Editor-in-chief,

We are grateful to the editor and reviewers for their time and constructive comments on our manuscript. We have incorporated all the comments and suggestions and here by to submit a revised version of the manuscript using track change and point by point response letter for further consideration in the journal. As per your request here are the lists of amendments.

**Journal Requirements:**

**Editor ’s comment**

Dear Editor,We are deeply grateful for your insightful advice and positive recognition of our work. We believe the manuscript has been significantly strengthened through the incorporation of your and the reviewers' valuable feedback. Our detailed point-by-point responses to your comments are provided below.

1. Please ensure that your manuscript meets PLOS ONE's style requirements, including those for file naming. The PLOS ONE style templates can be found at

**Response to editor’s comment:** Thank you so much for your guidance, we have revised our manuscript based on the PLOSE ONE journal style template and submission guideline.

**Editor ’s comment**

2. Your ethics statement should only appear in the Methods section of your manuscript. If your ethics statement is written in any section besides the Methods, please move it to the Methods section and delete it from any other section.

**Response to editor’s comment:** Thank you so much for your suggestion, the ethics statement is included in the methods section following data processing and analysis.

**Editor ’s comment**

3. We note that you have indicated that there are restrictions to data sharing for this study. For studies involving human research participant data or other sensitive data, we encourage authors to share de-identified or anonymized data. However, when data cannot be publicly shared for ethical reasons, we allow authors to make their data sets available upon request. For information on unacceptable data access restrictions, please see

**Response to editor’s comment:** Thank you so much for your comment, as far as our data set is concerned, there is no any restrictions, and we are able to submit the minimally anonymized data set as an additional file.

**Editor ’s comment**

4. Your abstract cannot contain citations. Please only include citations in the body text of the manuscript, and ensure that they remain in ascending numerical order on first mention.

**Response to editor’s comment:** Thank you so much for your suggestion, but our abstract does not include citations. We apologize if the numbers we included in the method section, which shows the vicarious trauma score as low risk (0–14), moderate risk (15–21), high risk (22-28), and extremely high risk (29–35), are making confusion.

**Editor’s comment**

5. Figure 1 in your submission contain [map/satellite] images, which may be copyrighted. All PLOS content is published under the Creative Commons Attribution License (CC BY 4.0). We require you to either (1) present written permission from the copyright holder to publish these figures specifically under the CC BY 4.0 license, or (2) remove the figures from your submission.

**Response to editor’s comment:** Thank you so much for your comment, we discussed the choices you mentioned in comment number five, and we decided to delete figure one from the manuscript.

**Editor’s comment**

6. We note you have included a table to which you do not refer in the text of your manuscript. Please ensure that you refer to Table 2 in your text; if accepted, production will need this reference to link the reader to the Table.

**Response to editor’s comment:** Thank you so much for your comment, Table 2 has been included on the manuscript at the end of the text description, where to view at the end sentence of page 9.

**Additional Editor specific Comments:**

**Abstract**

**Editor’s comment:** Avoid third-person writing “the”

**Response to editor’s comment:** Thank you so much for your suggestion, we have revised the article “the” and made some modifications
**Editor’s comment:** VT seems difficult to understand so it is better to define it in the abstract as well as in one sentence.

**Response to editor’s comment:** Thank you so much for your suggestion, we have revised the abstract, we wrote in full sentence as vicarious trauma and add one sentence “Vicarious trauma refers to harmful changes that occur in professionals’ views of themselves and/or others as a result of deep empathic engagement and repeated exposure to details of trauma survivor”.

**Editor’s comment:** “we estimated “third person writing the whole sentence needs rephrasing

**Response to editor’s comment:** Thank you so much for your comment, we tried to revise the whole sentence and make an edition.

**Editor’s comment:** The burden of war and its impact especially on those with health providers, what is the need to do especially health care workers

**Response to editor’s comment:** Thank you so much for your comment, in conflict zones, humanitarian agencies emphasize the critical need to safeguard healthcare workers so they can continue their life-saving work, International Humanitarian Law (IHL) and respect for healthcare; agencies like the ICRC, WHO, and Safeguarding Health in Conflict Coalition (SHCC) stress that healthcare workers and facilities are protected under the Geneva Conventions and additional protocols. Attacks against them are war crimes.

**Editor’s comment:** I would put AOR of the factors significantly associated

**Response to editor’s comment:** Thank you so much for your suggestion; we put the value of AOR for the factors significantly associated.

**Introduction**

**Editor’s comment:** Line 3-5 can you quantify the material or human damage with reference
**Response to editor’s comment:** Thank you so much for your suggestion; the total economic cost of the war-related looting or vandalism in monetary terms was more than $3.78 billion, and the damage to the economic value in monetary terms was more than $2.31 billion. Meanwhile, the direct economic loss to the health system in monetary terms was more than $511 million. According to this assessment, 514 (80.6%) health posts, 153 (73.6%) health centers, 16 (80%) primary hospitals, 10 (83.3%) general hospitals, and 2 (100%) specialized hospitals were damaged and/or vandalized either fully or partially due to the war. Reference kindly look at the articles “damage to the public health system caused by war-related looting or vandalism in the Tigray region of Northern Ethiopia” cited as (Gufue ZH et al., Front. Public Health 12:1271028. doi: 10.3389/fpubh.2024.1271028).

**Editor’s comment:** Citation for the “deliberate targeting of war on health care system causing disruption

**Response to editor’s comment:** Thank you so much for your comment; we put the reference for this sentence which was paraphrased from the sentence“Many have reported that the deliberate destruction, vandalisation and looting of the entire health system have been the hallmarks of the ongoing conflict” published by Hailay Gesesew and his colleagues cited as (Gesesew H, Berhane K, Siraj ES, et al., BMJ Global Health 2021;6:e007328. doi:10.1136/ bmjgh-2021-007328).

**Editor’s comment:**

- Paragraph 3; why healthcare professionals; are at risk needs rephrasing and synthesis,

• Rather than listing all studies to connect ideas and flow in paragraphs 3 and 4
• First discussing issues in Western then Africa rather than shifting back and forth

**Response to editor’s comment:** Thank you so much for your comment; we rewrite paragraph 3 and 4, where to look paragraph 3 on the “revised manuscript with track and manuscript” attached.

**Methodology**

**Editor’s comment:** You need to describe the desire why you used p 50 % inside the text and design effect 2; need justification.

**Response to editor’s comment:** Thank you so much for your comment; we used p 50% because there were no related studies in Ethiopia and taking prevalence from other countries might not represent the situation in Tigray. Thus, we had used p 50%. Regarding the design effect of 2, as a rule of thumb design effect of 2 or 1.5 is frequently used in sampling, especially for cluster, multistage, and stratified designs because of their stages and intracluster correlation, to provide a practical way to inflate the sample size and account for the potential loss of precision compared to simple random sampling. However, it's essential to recognize its limitations and strive for more precise estimates of the design effect whenever possible.

**Editor’s comment:** If you put a diagram and the specific numbers when doing multi-stage sampling and proportional allocation

**Response to editor’s comment:** Thank you so much for your comment; we put the diagrammatic presentation of sampling technique and uploaded as Figure 1.

**Editor’s comment:** What other tools or factors are assed other than VT, which is not put in the methodology

**Response to editor’s comment:** Thank you so much for your suggestion; we add on the methodology all the rest factors such as sociodemographic, and work area related characteristics. **Editor’s comment:** Why do you use a p-value 0.05 for bivariate logistic regression and put the justification as well

**Response to editor’s comment:** Thank you so much for your suggestion; recognizing the absence of a definitive threshold for transitioning variables from bivariate to multivariate analysis, our study, leveraging a substantial sample size that inherently yielded a high variable-to-case ratio, strategically advanced all variables exhibiting statistical significance (p < 0.05) in the bivariate logistic regression to the subsequent multivariate model. This approach was deliberately employed to rigorously control for potential confounding influences and to examine the relationship between independent and dependent variables.

**Result**

**Editor’s comment:** You can also put the reliability of the tools of outcome and validation in Ethiopia.

**Response to editor’s comment:** Thank you so much for your comment; to ensure the robustness of our data collection, the reliability of the tool was rigorously assessed within the Ethiopian setting. This evaluation, conducted as part of our methodological approach, demonstrated strong internal consistency, evidenced by a Cronbach's alpha of 0.856 and we had putted at the “data quality control section at the end of the paragraph.

**Editor’s comment:** The footnotes a, b,c are confusing as you only used p-value 0.05 as statically significant in your methodology.

**Response to editor’s comment:** Thank you so much for your comment; the statistical significance of the identified factors is denoted by superscript letters, where 'a' indicates a p-value ranging from 0.05 to 0.01, 'b' signifies a p-value between 0.01 and 0.001, and 'c' represents a highly significant p-value of less than 0.001. If seems not important to show the level of significance we can remove.

**Editor’s comment:** Why you did not asses the type of traumatic content disclosed to health providers

**Response to editor’s comment:** Thank you so much for your comment; during the development of our proposal, we made a conscious decision to avoid directly questioning healthcare providers about the specific traumas they had experienced. This decision was based on several factors: (1) the potential for multiple trauma exposure would make it difficult to pinpoint the source of vicarious trauma; (2) the varied trauma histories of patients could obscure the link to specific instances of vicarious trauma; and (3) eliciting detailed accounts of past traumas could inadvertently exacerbate vicarious trauma symptoms through recall and reflection during the data collection process.

**Editor’s comment:** Consequently, medical personnel were forced to treat a high volume of patients with the potentially dangerous physical and psychological effects of the conflict on a regular basis. Another explanation could be that many medical experts and dangerous medical supplies needed for treatment and therapy have been forced to leave underfunded, damaged, and robbed healthcare institutions. This places a great deal of strain on the remaining employees, who are already probably overburdened and worn out, making them even more susceptible to vicarious stress. The Tigray war, which was characterized by ethnic violence and the targeting of civilians, which medical professionals may see personally while tending to patients, this could be because the victims have experienced personal loss, have lost loved ones or families, or have been uprooted themselves. This may exacerbate their inability to detach themselves from their traumatized patients and heighten their sense of helplessness and hopelessness.” This concept needs to be back up by citation and references

**Response to editor’s comment:** Thank you so much for your comment; based on the authors' direct experiences during the war, and supported by the literature cited in references (19, 20), this paragraph outlines the difficult circumstances faced for healthcare providers.

**Editor’s comment:** The discussion needs to be improved as it does not compare or give appropriate citations for the explanation of the discussion points.

**Response to editor’s comment:** Thank you so much for your comment; we revised the discussion and corrected on the manuscript.

**Editor’s comment:** I would add a specific and better recommendation.

**Response to editor’s comment:** Thank you so much for your comment; we also tried to revise the recommendation.

**Response to Reviewer #1**

Reviewer suggestion: I hereby submit a review report of the manuscript, entitled ‘War related -Vicarious Trauma among Healthcare providers in the War-Torn Tigray, Northern Ethiopia”. I acknowledge the efforts of the authors for performing a good job and writing evidence, which is highly needed. Since, it needs further amendment, I suggested to be accepted with Major revision. I believe, this manuscript will be very helpful in passing better evidence for decision making in rebuilding the human resource development.

**Response to reviewer suggestion:** Dear Reviewer, we are deeply grateful for your insightful advice and positive acknowledgment of our work. We believe the manuscript has been significantly strengthened through the incorporation of all the valuable feedback from you and the editor. Our point-by-point responses to your specific comments are detailed below.

**Reviewer comment:** For the sake of simplicity, it would be good to create line numbers of the manuscript, which would be easier for the authors to trace the comments and revised changes.

**Response to reviewer comment:** Thank you so much for your suggestion; we put line number on the manuscript.

**Abstract**

**Reviewer comment:** On the method section of the abstract a statement is described as “VT was assessed using a 7-item standard tool with likert scale…”, a phrase in a new line statement shall be written with full words of the phrase, otherwise, authors are advised to write Vicarious Trauma instead of VT

**Response to reviewer comment:** We appreciate your comment and have used full words throughout the abstract, particularly on new lines.

**Reviewer comment:** On the second line of the result section of the abstract, authors need to rephrase the “was found to be”.

**Response to reviewer comment:** We are grateful for your comment and have revised the phrase based on your suggestion.

**Reviewer comment:** The conclusion of the abstract seems overlooked.

**Response to reviewer comment:** Thank you so much for your comment; based on your input, we undertook a revision of the conclusion.

**Introduction**

**Reviewer comment:** On the first and second paragraphs: Authors have verified about the war in Tigray and its effects on the vulnerability of the health workers referring three peer reviewed articles. Ample literature written by humanitarian organizations have proven the health workforce traumatic experiences, so authors are requested to justify their statements from the perspective of humanitarian agencies.

**Response to reviewer comment:** Thank you so much for your comment; we have added some evidence from the humanitarian agencies, where to find them the whole paragraph 2.

**Reviewer comment:** Third and fourth paragraph, the piece of evidences from Gaza, Poland, Eastern Maynemar, Kenya, USA, Japan and findings from University of Ottawa needs to be consolidated, refined and synthesized.

**Response to reviewer comment:** Thank you so much for your comment; we've revised and rewritten the content you were commented about. You can find the updated version in the fourth paragraph of the attached "revised manuscript with track change and manuscript” attached.

**Reviewer comment:** Though large number of health workforce population and civilians suffer in SSA countries due to war and conflict, the experiences and prevalence of vicarious trauma in sub Saharan African countries seem to be overlooked.

**Response to reviewer comment:** We appreciate your comment; we looked for articles about vicarious trauma among health care professionals in Africa and sub-Saharan Africa, but we were unable to find that directly related to the topic. These articles mostly discussed destruction in terms of materials, human lives, services, economics, and displaced millions of people, compassion fatigue, and burnout. If we do find any, we would be happy to add them.

**Reviewer comment:** On top of these comments, authors are advised to revise the flow and coherence of the paragraphs in the introduction section. It would be advisable to follow the writing style from global to local and general to specific

**Response to reviewer comment:** Thank you so much and really appreciate your comment; we changed the introduction section, moving the problem statement in particular from global to specific.

**Reviewer comment:** Last paragraph of the introduction section: ……authors are advised to revise the phrase; instead of “not investigated” “the scope of the problem is not adequately investigated” might be an option. It would also be good to rephrase the valid evidence stated by “Ayder referral hospital”.
**Response to reviewer comment:** We really glad and appreciate your comment; we made the necessary arrangements and reworded the last paragraph.

**Methods and materials**

**Reviewer comment:** Study area**;** It is good that authors noted the number of health facilities and health workforce before the war. On top of the information given the categories of the health workers, their speciality and the development of the health workforce and its contribution on the health outcome need to be highlighted.

**Response to reviewer comment:** Thank you so much for your comment; we add the category and specialty of the healthcare professional.

**Reviewer comment:** Study design; The design is not only for the purpose of recruiting healthcare providers. It works for conducting the overall study. Authors are advised to omit the phrase “recruiting healthcare providers

**Response to reviewer comment:** Thank you so much and much appreciated for your comment; we omit the phrase and edited it.

**Reviewer comment:**Sample Size and Sampling Procedure; What was the reason for authors to choose simple random sampling technique after using multistage stratified sampling technique, while systematic random sampling technique is the easiest technique? It is from curiosity.
 **Response to reviewer comment:** Thank you so much and great appreciate for your comment; indeed systematic sampling technique is the best and ideal approach for such study having with list (frame), in this case due to the war most of the healthcare facilities had looted/destructed including their documents and over all files. Thus, we choose simple random sampling technique.

**Reviewer comment:** Authors noted “Twelve health professionals fluent in the local language (Tigrigna) were recruited.

**Response to reviewer comment:** Thank you so much for your suggestion; we have revised this sentence.

**Results**

**Reviewer comment:** Socio-demographic characteristics of study participants
The first line statement reads “From the total of 2374 sampled healthcare providers, 2366 participants were included in the study”, I suggest to be corrected as “of 2374 participants, 2366 participated in the study” Authors are advised to revise their reports to be concise.
**Response to reviewer comment:** Thank you so much for your suggestion; we tried to revise the reports.

**Reviewer comment:** The first paragraph of the result section is with redundant phrases “two third is stated four times in a single paragraph”. Authors are advised to avoid redundant phrases and rephrase their result statements with eye-catching ideas to readers.

**Response to reviewer comment:** We are highly appreciated to your comment; based on your suggestion, we have revised and rewrites the paragraph.

**Reviewer comment:** Besides authors interpreted “Nearly two-thirds of the study participants were from the Mekelle and Central zones” that mismatches with the figure on table 1 which is calculated as 26.7% for Mekelle and 15% for Central in total 41.7%. Authors are advised to make correction of the aforementioned result statement.

**Response to reviewer comment:** We thank you immensely and greatly appreciated for your valuable feedback; we have carefully corrected the identified mismatch in the sample sizes for Mekelle (701, 29.7%) and Central (631, 26.6%), totaling 56.3%. You can find these corrections detailed in Figure 1 (sampling procedure), Table 1, and Table 2.

**Reviewer comment:** What standard do you for perceived economic classification?

**Response to reviewer comment:** Thank you so much for your comment; while recognizing that objective measures like the wealth index are standard for economic classification, our study utilized a subjective assessment. This approach captured each individual's personal perception and interpretation of their own economic standing, categorized as rich, medium, poor, unable to classify, or "I do not want to talk."

**Reviewer comment:** In your result section table 2, the result of the odds ratio 0.7 [0.5, 0.9] by zone showed the protective effect of vicarious trauma among health workers in south East zone, do you have any clarification? Can you see back your data again for possible correction of the analysis?

**Response to reviewer comment:** Thank you so much for your comment; we thoroughly reviewed the data and confirmed the reported result. Therefore, the interpretation that the south east zone was 30% less likely to develop vicarious trauma compared to the Mekelle zone stands. This suggests a lower prevalence of vicarious trauma in the south east, though it was still present. A potential explanation for this difference could be its greater distance from the conflict-affected border areas of Eritrea and the Amhara region and number of massacres, rape, number of survivors.

**Discussion**
**Reviewer comment:** In the first paragraph of the discussion the word those is repeatedly stated, so authors are requested to avoid redundant words and revise the wording. The comparison of similarities and variations in the discussion section is more skewed to the Western countries (USA, Europe and Canada). In order to make careful comparisons with sub-Saharan African countries, Authors need to revisit the discussion section.
**Response to reviewer comment:** Thank you so much for your comment; we have tried to avoid redundant words as much as possible and revised the discussion.

**Reviewer comment:** Many of the justifications stated on the discussion section lack references. Similar justifications are documented in various reports by humanitarian organizations including MSF, OCHA, ICRC and World Peace foundation led by Professor Alex Alex de Waal, so authors are advised to use the references for their justifications

**Response to reviewer comment:** We are so glad and appreciated to your comments; we have revised and add points from humanitarian organization reports. Where to find paragraph 3 discussion points.

**Reviewer comment:** Authors are duly requested to revisit the discussion section for further elaboration and amendment

**Response to reviewer comment:** Thank you so much for your suggestion; we have revised the discussion part and correction is made accordingly.

**Reviewer comment:** For the betterment of the manuscript, language revision is highly needed by native speakers.

**Response to reviewer comment:** We greatly appreciated for your comment; we tried to revise and edited the language and any typo errors.

**Response to Reviewer #2:**

**Reviewer suggestion**: The title is very interesting and timely to improve the healthcare service. However, the following comments needs your attention
**Response to reviewer suggestion:** Dear Reviewer, we are deeply grateful for your insightful advice and positive acknowledgment of our work. We believe the manuscript has been significantly strengthened through the incorporation of all the valuable feedback from you and the editor. Our point-by-point responses to your specific comments are detailed below.

**General comments:**

**Reviewer comment:** There is gloss editorial problem, less contextualized (context is war and war-related), the way research question is explored is not clear
**Response to reviewer comment:** Thank you so much for your general comment; we have revised and edited the manuscript solely and tried to contextualized with war-related articles including from the humanitarian organization mentioned about war. In addition, we have tried to describe and need to investigate the aftermath consequence of war on healthcare providers as a research question. Where to find on the attached manuscript with track change and manuscript.

**Abstract**

**Reviewer comment:** Background: Indicate clear gap

**Response to reviewer comment:** We greatly appreciated for your comment; we tried to show the gap on back ground section of the abstract on the revised manuscript.

**Reviewer comment:** Method does not indicate study area

**Response to reviewer comment:** Thank you so much for your comment; based on your impute we add the study area.

**Reviewer comment:** Result: show the p values for the factors

**Response to reviewer comment:** Thank you so much for your comment; we have put the p-value for the factors on the abstract section.

**Reviewer comment:** Conclusion: disregard the conclusion about risk factors

**Response to reviewer comment:** We highly appreciate your suggestion; we have tried to summarize the factors and add on conclusion part.

**Introduction**
**Reviewer comment:** Look the detailed comments in the document

**Response to reviewer comment:** We are very glad on your comments that you putted at the main document and we have revised based on your valuable comments.

**Method**

**Reviewer comment:** The study setting is not clear... Conditions during the war are not indicated

**Response to reviewer comment:** Thank you so much and great appreciated for your comment; we have add the situation of the health facility during the study period (immediately after the war). Where to find, kindly look at the revised manuscript on methods and material part, study area section.

**Reviewer comment:** Show that your study takes place in health facilities under the study area and setting

**Response to reviewer comment:** Thank you so much for your comment; we have indicated on the study area as the study is conducted at health facilities.

**Reviewer comment:** Specify your study population (if any inclusions and exclusions)

**Response to reviewer comment:** We really appreciated on your comment; we have add study population and who were included with the study design section.

**Reviewer comment:** Sampling procedure like stratification and proportional allocation should be clear (look the comments in detail in the document)

**Response to reviewer comment:** Thank you so much for your nice comment; we stratified the health facilities as; Referral hospitals, general hospitals, primary hospitals, health centers, and health posts. Where to find on the revised attached as figure 1 (schematic presentation of sampling procedure). Due to damaged/looted documentation of some health facilities, we have used simple random sampling technique instead of systematic sampling technique. Concerning the approach, first we proportionally allocated the sample size to their specific health facilities, next, based on the sample allocated to the specific health facility, we proportionally allocated again to each department of that facility. Finally, we have randomized from each department/units based on their respective sample. Hence, we did not considered for specific department based on their expected exposure to survivors because the Tigray war is probably different in its nature, meaning that all health care providers were directly or indirectly exposed to survivors. As solution departments was used as one independent factor and we did not find significantly associated with vicarious trauma.

**Reviewer comment:** How frequent? Do you think all selected health facilities are connected to the server for monitoring? Consider access to network and the likes

**Response to reviewer comment:** Thank you so much for your comment; our data manager had been checked daily at the end of the data collection and all facilities were linked to the server and we used GPS coordination which is easily connected using the ODK tool.

**Reviewer comment:** Analysis method... way of reporting the OMLR... Comment in the document

**Response to reviewer comment:** We much appreciated for your comment; we have checked the assumptions for ordinal logistic regression models during worked on the OMLR.

**Reviewer comment:** Inclusion of the zones for analysis... Concerns look comment

**Response to reviewer comment:** Thank you so much for your comment; we had looked the result variation among the zones. Hence, the result, in southeastern zone 0.7 (0.5,0.9) indicated they are suffered still for VT but 30% less likely having develop VT as compared to Mekelle zone. Thus, does not mean healthcare workers were free/protective from developing VT.

**Discussion**
**Reviewer comment:** Need a huge work, Justifications provided are not satisfactory and not supported by evidence, Implications are not indicated, All the narrative in the discussion should be based on scientific merit.

**Response to reviewer comment:** We are delighted and deeply appreciated your comment; we have revised the discussion part and add evidences including from the witness of humanitarian agencies.

**Reviewer comment:** Strength and limitation... very shallow

**Response to reviewer comment:** Thank you so much for your comment; we tried to revise and rewritten.

**Conclusion**;

**Reviewer comment:** Has to emanate from the findings, Conclusion: not repeating the result

**Response to reviewer comment:** We greatly appreciated to your comment; we have revise and edited the conclusion part based on your directions.

**Response to Reviewer #3**

**Reviewer suggestion:** First of all, I would like to say thank you dear plos one journal organizer for inviting me to this valuable manuscript and thanks Authors for well-organized and systematically writing the manuscript. I moved through all parts of the manuscript and no doubt has been seen. It has acceptable comment and no more major errors are seen. Regards!

**Response to reviewer suggestion:** Dear reviewer, we are delighted and greatly appreciated, thank you so much for your kind words, acknowledge, and recognized and give credit for our work**.**

**Response to Reviewer #4**

Dear reviewer we are much appreciated and delighted for your strong advise and give credit for our work, though the paper become more strong after incorporating all the reviewers and editor comments. Here below are point-by-point response for your comments.

**Reviewer comment 1:** In the title, the phrase “War related-Vicarious Trauma” should be: “War-Related Vicarious Trauma

**Response to reviewer comment:** Thank you so much for your comment; we accepted your input and edited the title.

**Reviewer comment 2:** Please change “It has costed the physical, mental, economic, and psychological health of the people in Tigray in general and the healthcare providers in particular” to “It has taken a toll the physical, mental, economic, and psychological health of the people in Tigray in general and the healthcare providers in particular

**Response to reviewer comment:** We greatly appreciated to your comment; we accepted your comment and replaced it.

**Reviewer comment 3:** Please clarify the sentence: “The effect of conflicts provided a prominent example of this experience”

**Response to reviewer comment:** Thank you so much for your comment; we tried to clarify and rephrased the sentence. It means “the impact of war offered a notable illustration of a traumatic event for healthcare providers”.

**Reviewer comment 4:** For the sentence “A systematic review revealed that 56% of health care providers had developed secondary trauma (10)” please clarify to be consistent with the other examples cited in this paragraph by briefly identifying the work setting and affected group.
**Response to reviewer comment:** We are greatly appreciated to your comment; we revised and described based on their study settings and their professions.

**Reviewer comment 5:** Maynemar” should be “Myanmar”

**Response to reviewer comment:** Thank you so much for your comment; and apology for the typo error, now we have corrected.

**Reviewer comment 6:** Please change “Despite our personal observations and experiences with such trauma among healthcare providers, in the war-torn Tigray region, ..… to “Despite our personal observations and experiences with such trauma among healthcare providers in the war-torn Tigray region....

**Response to reviewer comment:** We much appreciated on your comment; we revised this phrase based on your comment.

**Reviewer comment 7:** Please define “health posts,” which is a term that may not be familiar to all readers

**Response to reviewer comment:** Thank you so much for your suggestion; we have put a definition for the term health post at the study area section.

**Reviewer comment 8:** Regarding the primary outcome measure, the “Crisis and Trauma Resource Institute's (CTRI) tool,” please specify in the text which tool was used (eg., title and if available the author(s), publisher, publication year, etc). Has the tool’s validity and reliabilty been measured and published in peer reviewed literature? If so, please cite using the publication information instead of the URL where it is found.

**Response to reviewer comment:** Thank you so much for your comment; this tool has been utilized by us, and we've noted similar applications in publications such as "Burnout, Compassion Fatigue and Vicarious Traumatic Experiences Among Social Workers: Emotional Regulation as a Protective Factor" by Hooria Azam et al. (2023) and "Vicarious trauma in mental health care providers" by Rosalinda R. et al. (2021).

**Reviewer comment 9:** Also regarding the primary outcome measure, for the sentence stating that “After extensive revision of the English questionnaire, the final English version was translated into the local language by language experts” please briefly describe or summarize the descriptions. What needed revision?

**Response to reviewer comment:** We appreciated on your comment; we make summarize and rephrased the sentence. This sentence is not mainly with outcome measurements rather it indicates with the tool of socio demographic characteristics and related to the healthcare’s work environment.

**Reviewer comment 10:** In the sentence “Data collection location, accuracy, and completeness were monitored by the data manager by looking at the server-centered,” the meaning of the phrase “looking at the server-centered” is unclear. Please clarify.

**Response to reviewer comment:** Thank you so much for your comment; we tried to rephrased the sentences on the revised manuscript as “Data collection location, accuracy, and completeness were monitored by the data manager throughout the data work flow using the ODK data repository.

**Reviewer comment 11:**For the sentence “The reliability of the item was measured by a Cronbach's alpha that ranged from 0.835 to 0.856” please clarify “The reliability of the item was measured by a Cronbach's alpha that ranged from 0.835 to 0.856.” Is this inter-rater reliability, something else

**Response to reviewer comment:** Thank you so much for your comment; this inter-rater reliability indicated the range value of Cronbach alpha for each item of the outcome measurement, indicates strong internal consistency of the measuremnts.

**Reviewer comment 12:** ……the fact that the ordinal logistic regression variables were derived empirically rather than from a pre-specified hypothesis means that the on average, 5% of the significance could be explained by Type I error. This should be acknowledged as a limitation of the study. It should also be clarified that the estimated sample size and implied power analysis relate to measuring significance in the primary outcome of vicarious trauma only, not to the regression model (assuming this is the case; if not, please clarify).

**Response to reviewer comment:** We sincerely appreciate to your comment; we have acknowledged and incorporated your first point as a limitation of the study. The second issue you raised is addressed and described in the data processing and analysis section of the Methods. **Reviewer comment 13:** The meaning of “than” is not clear in the sentence “When we report our results of multivariable ordinal logistic regression, we used the term ‘higher level vicarious trauma,’which means extreme high than combined low, medium, and high…….;Instead of “than” do you mean “versus” (or an equivalent term such as “rather than” or “as opposed to”)?

**Response to reviewer comment:** Thank you so much for you suggestion; we have replaced than by ‘versus’ or ‘rather than’.

**Reviewer comment 14:** By “orthodox religion” Do you mean the Christian Orthodox religion? Please clarify.

**Response to reviewer comment:** Thank you so much for your comment; yes, we edited the term as Christian Orthodox

**Reviewer comment 15:** Regarding“The participants had a median (IQR) work experience of 8.0 (5.0, 12.0) years and a median annual income (IQR) of 8017 Ethiopian Birr (range:7071.0–9056.0)” is this correct? I believe 7071.0–9056.0 EthBirr is equivalent to US$55.05-70.51, while the 2023 Gross Domestic Product per capita in Ethiopia was last recorded at US$890.35 US. Please confirm this is correct both in the foregoing sentence and also in Table1.

**Response to reviewer comment:** We greatly appreciate your comment; the reported median salary and interquartile range (IQR) reflect the current monthly earnings of the study participants. We did not include a comparison to the national standard for salary in this analysis.

**Reviewer comment 16:** Regarding “Table 2: Factors associated with VT…” I suggest the authors consider making this Table 3, and adding a separate Table 2 in which data are presented on the prevalence of the different levels (low risk, moderate risk, high risk, and extreme high risk) of vicarious trauma in the sample as a whole. Alternatively, perhaps even better than a new Table 2 could be a new Figure 1 with this data represented graphically using a pie chart.
**Response to reviewer comment:** Thank you so much for your comment; regarding the presentation of vicarious trauma prevalence, we've illustrated this in Figure 2 of the attached file. While we considered presenting all non-significant factors in a separate table, we ultimately decided against it to maintain reader focus. Instead, Table 2 specifically highlights the frequencies of the significantly associated factors in relation to the levels of vicarious trauma, aiming for clarity and ease of understanding for the readers.

**Reviewer comment 17:** For the sentence “Another explanation could be that many medical experts….” please revise for clarity, eg: “Another explanation could be that underfunding of, damage to,… in medical experts and essential medical supplies needed for treatment.”

**Response to reviewer comment:** Thank you so much for your nice comment; we accepted your comments and rewritten the sentence.

**Reviewer comment 18:** For the sentence, “The Tigray war, which was characterized by ethnic violence and the targeting of civilians,….” please revise for clarity, eg: “The Tigray war was characterized by ethnic violence and the targeting of civilians. Medical professionals….. have been uprooted themselves may be re-traumatized when tending to patients who have also experienced such trauma.

**Response to reviewer comment:** We greatly appreciated for your comment; we revised and rephrased this sentence according your suggestion.

**Reviewer comment 19:** Please consider clarifying “This might mean healthcare providers working in these regions were exposed to more violence and trauma……., like the of mass massacre in Tsion on November 29, 2020,” eg., “This might be because healthcare providers working in these regions ….such as the of mass massacre in Tsion on November 29, 2020 by troops of Eritrean and Amhara Milasha.” Please also provide citation for this statement, eg: by human rights groups, journalists, etc.

**Response to reviewer comment:** We greatly appreciated for your comment; we have revised this sentence and supported by evidences from human right watch report.

**Reviewer comment 20:** Please clarify “This implication might indicate the life stage and responsibilities; this means care providers over 30 may have additional personal stressors like…...”Eg.“This might be because of increasing life stage responsibilities and associated personal stressors among care providers over 30 such as…..

**Response to reviewer comment:** We are greatly appreciated for your comment; we have revised the sentence and rewritten according to what you have suggested.

**Reviewer comment 21:** Regarding the sentence “It might also be because of their changes in coping mechanisms; this means that over time, coping strategies used earlier in a career may become less effective, and healthier coping mechanisms may not yet be developed, making individuals more vulnerable to the effects of vicarious trauma” it is not clear why coping would decrease with age.

**Response to reviewer comment:** We are highly appreciated for your comment; we tried to revise and supported by evidence though not directly related to VT the sentence stated as “it might also because of the age differences in stress and coping processes, this means that over time, older adults used less confrontive coping and more positive reappraisal and avoidance coping strategies.” Reference (Nascimento EM, et al., Pain in times of Covid-19: 2024)

**Reviewer comment 22:** Regarding the sentence “Limited personal time and reduced opportunities for self-care and emotional processing due to family commitments can exacerbate the impact of vicarious trauma” please either a.) provide a reference for this or b.) replace “can” with “might.”

**Response to reviewer comment:** We highly appreciated for your nice comment; we are accepted your suggestion and replace the word can with might.

**Reviewer comment 23:** Regarding “…but being hampered by the limited resources and seriousness of the war might lead ….., which can contribute to vicarious trauma.” Please either a.) provide a reference for this statement or b.) change “can contribute” to “might contribute.”
**Response to reviewer comment:** We are really appreciated your comment; similarly, to the above suggestion we admitted this comment too.

**Reviewer comment 24:** Please clarify “Additionally, it might be difficult to make decisions…..” Eg.,“Additionally, it might be difficult for managers to make decisions about….. and witnessing the overall impact of the war on healthcare delivery.”

**Response to reviewer comment:** Thank you so much for your comment; we have revised the sentence and corrected.

**Reviewer comment 25:** Please clarify “This might be because of the nature of the war, which was totally besiege and siege.” Eg.,“This might be because of the nature of the war, which involved working in areas under siege.”

**Response to reviewer comment:** We are delighted and great appreciated on your comment; we have revised and corrected the sentence.

**Reviewer comment 26:** Regarding the sentence, “This would mean healthcare professionals see more critically ill patients to reach their facilities. and those whose conditions worsen due to a lack of timely treatment,” wouldn’t this traumatize all healthcare workers equally irrespective of their distance from work?

**Response to reviewer comment:** We great appreciated on your comment; this sentence explain effectively addresses why those with longer commutes (sixty minutes and above) might report higher levels or severity of vicarious trauma, even though all healthcare workers in the region are undoubtedly exposed. Meaning that during their journey to their working area they might be exposure to the crisis such as witnessing critically ill patients and their struggling families en route to the facility (walking long distances, being carried) provides additional, direct visual encounters with suffering and the lack of access to timely care.

**Response to reviewer #5**

**Reviewer suggestion:** This study is important in examining the vicarious trauma on the Tigray War.
**Response to reviewer suggestion:** Dear reviewer, we are deeply grateful for your kind appreciation and recognition of our work. Thank you so much for your encouraging words and for acknowledging our efforts.

**Some concerns emerged.**
**Reviewer comment:** Socio-demographic characteristics of study participants is better to be simple using Table 1.

**Response to reviewer comment:** Thank you so much for your comment; sure, we understand your suggestion to simplify it as Table 1. However, to enhance clarity and facilitate easier understanding for readers, we opted to provide a descriptive sentence alongside Table 1.

**Reviewer comment:** The definition of the healthcare providers is better to be filled in the methods.

**Response to reviewer comment:** We sincerely appreciate your suggestion and have incorporated the definition of healthcare providers into the Methods section as you recommended. Thank you for your valuable input.

**Reviewer comment:** In the section of the factors Associated with Vicarious Trauma, on the aim of the analysis, what reason did the authors include the eight categories in the multivariable ordinal logistic regression mode?

**Response to reviewer comment:** We greatly appreciate your insightful comment; following bivariate ordinal logistic regression analysis of numerous independent variables against the outcome variable (vicarious trauma), the eight factors (categories) exhibiting a p-value < 0.05 were subsequently included in the multivariable ordinal logistic regression model. This step was taken to control for potential confounding effects, and this methodological approach has been clearly outlined in the Data Processing and Analysis section of the Methods.

**Reviewer comment:** Are the explanations of the results is overlapping to the table 2

**Response to reviewer comment:** Thank you so much for your comment; yes, the result is tried to explained or clarify on significantly associated variables, this was done to enhance readability and comprehension for future readers and researchers who utilize this paper post-publication. The accompanying table serves as a concise summary of these detailed findings, which we believe is readily understandable for those directly involved with the research.
