# Supplementary material for: War-related vicarious trauma among healthcare providers in the war-torn tigray, Northern Ethiopia
Source: PLoS One. 2026 Mar 4;21(3):e0340978. doi: 10.1371/journal.pone.0340978 (PMC12959660; doi:10.1371/journal.pone.0340978)
Supplement: Attachment — Submitted filename: Re-review of Vicarious trauma manuscript PLOS One.docx [file pone.0340978.s005.docx]

**May, 31^st^, 2025**

**Dear Academic Editor and authors**

**I am very glad to re-review the manuscript “War-related Vicarious Trauma among Healthcare providers in the War-Torn Tigray, Northern Ethiopia”**

I re-reviewed the revised manuscript and it is well written except few typographic and editorial errors. The revised version of the manuscript showed much improvement. Since the evidence is highly needed for decision making and restoration of the human resource development, I suggest this revised manuscript to be accepted after the minor errors are corrected.

**Minor comments that need to be considered are below:**

**Line 41:** Authors need to edit word spacing.

**Line 51-52:** Method of the abstract section: Authors wrote: “Statistical significance was reported whenever the 95% confidence interval for odds ratio **did not include one** and the p-value was less than 0.05”. This statement contradicts with their finding on line 235-236 Table 2: Factors associated with vicarious trauma among healthcare providers in Tigray; for instance in table 2, the variable age in years **“age group 20-29 is reference, then the vicarious trauma for the age groups 30-39, 40-49 and ≥50 showed results of AOR and CI 1.3 [1.1, 1.6], 1.5 [1.1, 2.0] and 2.3 [1.7, 3.0]. In this case the confidence intervals include 1 which contradicts to the statement reported by authors. Therefore, Authors need to reconsider their statement related to statistical significance based on the valid and scientific description.**

**Line 95 and 99:** Authors need to edit word spacing.

Authors wrote sometimes VC in abbreviation and others in full words; please **check Line 98, 100, 101, 104, 105, 110. Authors are advised to be consistent in writing the phrase.**

**Line 114:** Authors need to state “health post” in plural form as **“health posts”**.

**Line 199-200:** Authors stated: 66.1%, of the 200 respondents were living with their parents. It is uncommon to start with numbers in the new line statement, **so authors are advised to rewrite the statement: of the 200 respondents, two third/or 66.1% were living with their parents.**

**Line 328:** Authors wrote “**………………**consequence of the Tigary war among healthcare **professional”. The bolded word should be written in plural form. “professionals”**

**Reviewer – Dr. Tesfay Gebregzabher Gebrehiwet**
