# Supplementary material for: War-related vicarious trauma among healthcare providers in the war-torn tigray, Northern Ethiopia
Source: PLoS One. 2026 Mar 4;21(3):e0340978. doi: 10.1371/journal.pone.0340978 (PMC12959660; doi:10.1371/journal.pone.0340978)
Supplement: Attachment — Submitted filename: Response to reviewers second.docx [file pone.0340978.s007.docx]

**Responses to editor and reviewers’**

We are writing to express our sincere gratitude to both the editor and the reviewers for your invaluable time, insightful comments, and the positive reception of our previous responses. Your feedback has been instrumental in significantly enhancing the quality of our manuscript and reached at this stage. We have carefully incorporated all the additional comments and suggestions you provided. Accordingly, we are pleased to resubmit a revised version of our manuscript and manuscript with tracked changes for your easy reference, along with a detailed point-by-point response letter addressing each comment. As requested, a comprehensive list of all amendments is included for your further consideration. We genuinely appreciate your continued support. Thank you so much.

**Journal Requirements:**

Dear Editor, We are deeply grateful for your valuable advice and positive assessment of our manuscript. We feel the revisions, based on your and the reviewers' feedback, have significantly improved the work. Below, you will find our point-by-point responses to your few comments. We're very grateful for your detailed and insightful advice.

**Editor’s comment**

Please review your reference list to ensure that it is complete and correct. If you have cited papers that have been retracted, please include the rationale for doing so in the manuscript text, or remove these references and replace them with relevant current references.

**Response to editor’s comment:** Thank you so much for giving helpful suggestion. We have incorporated those references cited within the text.

**Additional Editor Comments (if provided):**

**Editor’s comment**

Thank you for revising your paper with comments and suggestions. However, these areas need further editing.

**Response to editor’s suggestion:** We are immensely grateful for your thoughtful feedback and the significant encouragement you have given us on this paper. It means a great deal to us. We are enthusiastic, thank you in advance.

**Abstract**

**Editor’s comment**

The burden of war and its impact, especially on those with health providers, what is the need to do, especially for healthcare workers, interventions such as aftercare and addressing the vulnerable groups, it would be better to add relevant recommendations on the conclusions based on the findings

**Response to editor’s comment:** We are deeply appreciated for your insightful suggestion; we add some specific recommendation based on our findings. Thank you a lot.

**Introduction**

**Editor’s comment**

It is good that you put references, but on lines 3-5, can you quantify in numbers the material or human damage?

**Response to editor’s comment:** Thank you so much for your comment, we have add the sentence related to the material damage on the manuscript “The total economic cost of the war-related looting or vandalism in monetary terms was more than $3.78 billion, and the damage to the economic value in monetary terms was more than $2.31 billion’ (Gufue ZH, et al., April 5, 2024). **Editor’s comment**

Paragraph 3; why health care professionals are at risk needs rephrasing and synthesis and Rather than listing all studies to connect ideas and flow in paragraphs 3 and 4.

**Response to editor’s comment:** Thank you for your insightful comment and it's greatly appreciated. We have rephrased and rewritten paragraph 3, and critically interconnected the ideas and strengthened the flow between paragraphs 3 and 4. These changes are visible in both the manuscript and the manuscript with track-change we have attached.

**Editor’s comment:** First discussing issues in western then Africa, rather than shifting back and forth.

**Response to editor’s comment:** Thank you so much for your suggestion, we have revised and tried to put from global to local (Western to Africa).
**Editor’s comment:** More details on the war in Ethiopia and its impacts, before going to the scarcity of data

**Response to editor’s comment:** We are highly appreciated for your nice insightful, thank you so much. We have revised and add paragraph 5 before directly going to the scarcity of the data.

**Methodology**

**Editor’s comment:** You need to describe the reason why you used p 50 % inside the text, and the design effect 2; need justification; no justification in the revised version and why did you use p-value of 0.05 for bivariate logistic regression and put the justification as well.

**Response to editor’s comment:** We deeply appreciate your feedback and have now incorporated our responses into the manuscript. Thank you so much.

**Result**

**Editor’s comment:** The footnotes a, b, c, are confusing, as you only used p-value of 0.05 as statistically significant in your methodology

**Response to editor’s comment:** We appreciate your kind suggestion. To be consistent with the methodology section we have specified the level of statistical significance, we have removed the footnotes. Previously, we had only included the variables to show their level of significance.

**Editor’s comment:** Not including the type of traumatic limitations as well disclosed to health providers.

**Response to editor’s comment:** We truly appreciate your very insightful comment. As we have tried to mention within our study's limitations, information regarding the specific types and durations of trauma exposure were not included in this study.

**Editor’s comment:** The discussion needs to be improved as it does not compare or give appropriate citations for the explanation of the discussion points

**Response to editor’s comment:** We highly appreciated for your comment; we have tried to revise the discussion part.

**Editor’s comment:** It is good to add specific recommendations based on the findings
**Response to editor’s comment:** We greatly appreciated for your insightful comment; based on your suggestion we have revised the recommendation and add some recommendations addressed specific activities.

**Editor’s comment:** Thorough edition of grammar and spelling

**Response to editor’s comment:** Thank you so much for your comment; we have tried to edit the grammar and spelling errors support by online free grammar checkers like quillbot grammar checker.

**Response to Reviewer #1**

Reviewer suggestion: Dear Academic Editor and authors, I am very glad to re-review the manuscript “War-related Vicarious Trauma among Healthcare providers in the War-Torn Tigray, Northern Ethiopia” I re-reviewed the revised manuscript and it is well written except few typographic and editorial errors. The revised version of the manuscript showed much improvement. Since the evidence is highly needed for decision-making and restoration of the human resource development, I suggest this revised manuscript to be accepted after the minor errors are corrected.

**Response to reviewer suggestion:** Dear Reviewer, we greatly appreciated for your insightful advice and positive acknowledgment of our work. Thus, your comments were found much more importance to reach our manuscript this stage of improvement among with the Editors and other reviewers’ comment, thank you so much. Here below we put our point-by-point responses to your specific comments;

**Minor comments that need to be considered are below**:

**Reviewer comment:** Line 41: Authors need to edit word spacing.

**Response to reviewer comment:** Thank you so much for your suggestion; we have edited space thank you so much for nice input.

**Reviewer comment:** Line 51-52: Method of the abstract section: Authors wrote: “Statistical significance was reported whenever the 95% confidence interval for odds ratio did not include one and the p-value was less than 0.05”. This statement contradicts with their finding on line 235-236 Table 2: Factors associated with vicarious trauma among healthcare providers in Tigray; for instance in table 2, the variable age in years “age group 20-29 is reference, then the vicarious trauma for the age groups 30-39, 40-49 and >50 showed results of AOR and CI 1.3 [1.1, 1.6], 1.5 [1.1, 2.0] and 2.3 [1.7, 3.0]. In this case the confidence intervals include 1 which contradicts to the statement reported by authors. Therefore, Authors need to reconsider their statement related to statistical significance based on the valid and scientific description.
**Response to reviewer comment:** We really appreciate your insightful; we attempted to explain that the AOR 95% CI does not contain the null value, which is 1.0. Any value above this, including its decimal value of 1.05, 1.1, 1.2, etc., is considered significant. However, in order to prevent ambiguity, we have utilized the p-value to indicate the level of significance instead of using such a statement.

**Reviewer comment:** Line 95 and 99: Authors need to edit word spacing

**Response to reviewer comment:** We truly appreciate your suggestion. We have corrected this word space.

**Reviewer comment:** Authors wrote sometimes VT in abbreviation and others in full words; please check Line 98, 100, 101, 104, 105, 110. Authors are advised to be consistent in writing the phrase.
Line 114: Authors need to state “health post” in plural form as “health posts”.

**Response to reviewer comment:** We are happy by your insightful for your comment; based on your suggestion we have revised and edited the whole document to be consistent.

**Reviewer comment:** Line 199-200: Authors stated: 66.1%, of the 200 respondents were living with their parents. It is uncommon to start with numbers in the new line statement, so authors are advised to rewrite the statement: of the 200 respondents, two third/or 66.1% were living with their parents.

**Response to reviewer comment:** Thank you so much for your nice comment; we have revised the sentence as your direction.

**Reviewer comment:** Line 328: Authors wrote “………………consequence of the Tigary war among healthcare professional”. The bolded word should be written in plural form. “professionals”

**Response to reviewer comment:** Thank you so much for your suggestion, we have revised as you suggested.

**Response to Reviewer #4 and #5**

Dear reviewers, we greatly appreciated for your insightful comments and suggestion you have had given on the previous comment that help us to develop this improved manuscript. Thank you so much all.
