# Supplementary material for: War-related vicarious trauma among healthcare providers in the war-torn tigray, Northern Ethiopia
Source: PLoS One. 2026 Mar 4;21(3):e0340978. doi: 10.1371/journal.pone.0340978 (PMC12959660; doi:10.1371/journal.pone.0340978)
Supplement: Attachment — Submitted filename: Response_to__reviewers_auresp_3.docx [file pone.0340978.s008.docx]

**Responses to editor and reviewers’**

We are writing to express our deep gratitude to both the editor and the reviewers for your invaluable time, very insightful comments, and the positive reception of our previous responses. Dear all your feedback has been significantly building and enhancing the quality of our manuscript and reached at this stage. We have carefully incorporated all the additional comments and suggestions you provided. Accordingly, we are pleased to resubmit a revised version of our manuscript and manuscript with tracked changes for your easy reference, along with a detailed point-by-point rebuttal letter addressing each comment. As requested, a comprehensive list of amendments is included for your further consideration. We genuinely appreciate your continued support for this manuscript to reach at this stage. Thank you so much for your invaluable time and strong scientific comments, suggestions, and guidance.

**Response to Reviewer #6**

Reviewer suggestion: Thank you for writing this important article of vicarious trauma among Healthcare providers in the War-Torn Tigray, Northern Ethiopia. My comments are provided below.
**Response to reviewer suggestion:** Dear reviewer, we greatly appreciated for your kind words and positive acknowledgment of our work. Thus, your comments were found much more importance to reach our manuscript this stage of improvement among with the Editors and other reviewers’ comment, thank you so much for your insightful comments and suggestions you provided. Here below we put our point-by-point responses to your specific comments:

**Reviewer comment:** Check grammar on line 72-75.

**Response to reviewer comment:** Thank you so much and we greatly appreciated for your nice comment, we have revised the grammar of the above paragraph and edited.

**Reviewer comment:** Typo word "involvement" in line 89

**Response to reviewer comment:** We really appreciate your insightful comment; we have corrected the typo error and wrote as “involvement”.

**Reviewer comment:** Typo word "similarly" in line 103

**Response to reviewer comment:** We truly appreciate your nice comment. We have corrected this typo error as “similarly”

**Reviewer comment:** Line 157 - use it is instead of "it's" in formal writing

**Response to reviewer comment:** We greatly appreciated for your insightful comment; based on your comment we have edited and wrote as “it is”.

**Reviewer comment:** Methodology: there is no report of the validity value of the tool used **Response to reviewer comment:** Thank you in advance for your insightful comment. We have revised and added the validity report of our measurement tool. Where to look on the data quality control line 186-188

**Reviewer comment:** Typo word "prevalence" in line 236

**Response to reviewer comment:** Thank you so much for your, we are really very delighted for your comments and we have corrected and wrote as “prevalence”.

**Reviewer comment:** Sometimes the word "moderate" is used (line 237), sometimes the word "medium" is used (Table 2): make sure the term used is consistent.

**Response to reviewer comment:** Thank you so much for your insightful comment, we have revised and corrected as moderate instead of the word medium.

**Reviewer comment:** Improve grammar and comprehension in line 288-293

**Response to reviewer comment:** Thank you so much and greatly appreciated for your comment, we have rewrote and paraphrasing the paragraph. Where to look at the manuscript with track changes line 292-304.

**Reviewer comment:** Line 324-350 must have references to justify the claims

**Response to reviewer comment:** Thank you so much for your insightful comment. For this justification, we did not put a reference because this justification is the researchers’ point of view; just using their biological plausibility, they had stated the possible reason why families having larger family size had experienced more vicarious trauma.

**Reviewer comment:** There is no consistency in the format of the references, font sizes across the texts

**Response to reviewer comment:** Thank you so much for your great and insightful comment, we have revised and corrected the whole references its font size and other error.
